# Hyperpolarization-activated cation currents in medium-size dorsal root ganglion cells are involved in overactive bladder syndrome in rats

**DOI:** 10.1186/s12894-020-00698-z

**Published:** 2020-09-02

**Authors:** Chao Tan, Fei Yan, Li-Ping Yao, Jun-Ling Xing, Wei-Jun Qin, Kun Zhang, Guo-Jun Wu, Jian-Lin Yuan, Fei Liu

**Affiliations:** 1 Department of Urology, Xijing Hospital, Air Force Medical University, 15 Changle West Road, Xi’an, 710032 Shaanxi China; 2 Xijing Hospital of Digestive Diseases, Xijing Hospital, Air Force Medical University, Xi’an, Shaanxi China; 3 Institute of neuroscience, Air Force Medical University, Xi’an, Shaanxi China

**Keywords:** Overactive bladder, *I*_h_, Dorsal root ganglion, Hyperpolarization, ZD7288

## Abstract

**Background:**

To investigate the functions of the hyperpolarization-activated cation currents in medium-size dorsal root ganglion cells in a rat model of overactive bladder syndrome.

**Methods:**

Rats with OAB were screened using a urodynamic testing device. The whole-cell patch clamp technique was used to investigate changes in excitability and hyperpolarization-activated cation current (*I*_h_) of medium-size cells in the L6 dorsal root ganglia (DRG) of the OAB rats. Intrathecal injection of the specific *I*_h_ inhibitor ZD7288 was used to investigate changes of voiding function and *I*_h_ of medium-size cells in the L6 DRG.

**Results:**

The urinary bladder weight of the OAB rats was significantly increased (*p* < 0.01); However, 7 days after intrathecally administration of ZD7288 (2 μM), the weight of rat bladder was significantly reduced (*p* < 0.01). The excitability of the medium-size cells in the L6 DRG of the OAB rats was significantly increased, and the number of action potentials elicited by a 500 pA stimulus was also markedly increased. Furthermore, ZD7288 significantly reduced the excitability of the medium-size DRG cells. The medium-size cells in the DRG of the OAB rats had a significantly increased *I*_h_ current density, which was blocked by ZD7288.

**Conclusions:**

The *I*_h_ current density significantly increased in medium-size cells of the L6 DRG in the OAB model. A decrease of the *I*_h_ current was able to significantly improve the voiding function of the OAB rats, in addition to lowering their urinary bladder weight. Our finding suggested that the observed increase of *I*_h_ current in the medium-size DRG neurons might play an important role in the pathological processes of OAB.

## Background

Overactive bladder syndrome (OAB) is characterized by urinary urgency accompanied by frequent urination and nocturia, with or without urinary urge incontinence [1]. OAB severely affects the patients’ quality of life, since frequent urination can cause severe inconvenience, whereas long-term incontinence can lead to urinary tract infections, possibly leading to severe clinical complications affecting the patient’s physical and mental well-being [2]. Therefore, the micturition reflex which is subject to complex control from both the central and the peripheral nervous system, is attracting more attention. Anatomic studies have uncovered that the relevant afferent nerves mainly pass through the pelvic posterior, mainly from the L6 and S1 dorsal root ganglia (DRG) to the spinal cord, to trigger the micturition reflex [3]. According to the diameter of the neurons, the afferent DRG nerve fibers are classified as large, medium and small, which are further categorized into A_α/β_, A_δ_ and C-type fibers [4, 5]. The afferent nerves controlling of the urinary bladder include the myelinated A_δ_ and the unmyelinated C-type fibers [6]. The hyperpolarization-activated cyclic nucleotide-gated cation (HCN) currents (*I*_h_) have been reported to be expressed by many cell types, such as cardiac pacemaker cells and retinal photoreceptor cells, as well as central and peripheral nerve cells, including DRG cells.

It has been discovered that small- and medium-size nerve cells of the DRG which can control urinary bladder all possess *I*_h_ currents, and the *I*_h_ current of the medium-sized neurons is significantly higher than that of the small neurons [7]. This indicates that the *I*_h_ current is predominantly present in the medium-size DRG neurons. Even though the afferent neurons that control the bladder include C and A_δ_ type fibers, in normal physiological conditions, the A_δ_ fibers are solely responsible for the mechanical nociceptive reflex when the bladder expands to threshold pressure. This indicates that the medium-size neurons of the DRG play an important role in the micturition reflex. The *I*_h_ current activated by hyperpolarization of the HCN-gated channels has been shown to be involved in many important physiological functions, including the pacemaker function of the heart and brain as well as maintaining the resting membrane potential and cell excitability [8, 9].

Masuda and coworkers have discovered that ZD7288, a specific inhibitor of HCN-gated channels, can inhibit the *I*_h_ current in the medium-size cells that control the bladder, markedly increasing the duration of the afterhyperpolarization potential of an action potential [7]. This in turn implies that the *I*_h_ current influences the excitability of the medium-size DRG neurons that innervate the bladder. In order to find out the relationship between OAB and the *I*_h_ current in medium-size DRG neurons, this study was performed to characterize the electrophysiological properties of the *I*_h_ current in the medium-size cells of the L6 dorsal root ganglion in a rat model of overactive bladder, and to investigate its influence on the model animals’ urodynamics.

## Methods

### Experimental animals

Experiments were performed using adult female SD rats, aged 7–8 weeks, weighing 200–220 g, provided by the Air Force Medical University animal facility. Under standard experimental conditions, the animals were kept on a 12 h day and night cycle, at 25 °C, and were provided with rat chow (Allentown, USA) and water ad libitum. The rats were allowed to acclimatize to the laboratory environment for 1 week prior to starting the experiments. The experiments were approved by the Air Force University animal experiment ethics committee.

### Animal grouping and establishment of the rat model of overactive bladder syndrome

The investigators were blinded to group allocation during the experiments and analysis. The animals were fasted for 12 h before beginning the experiments. By random number table grouping method, the animals were randomly assigned to the sham intervention group, sham+saline group, sham+ZD7288 group, OAB group, OAB + saline group, and OAB + ZD7288 group. The sample size calculation of each group was based upon our pilot study. In our pilot study, we used 3 animals in each of the six groups and totally 18 rats. And we got the preliminary data from the urodynamic studies from the six experimental groups. And mean ± SD of Micturition Interval (Sec) in the six groups were 65.83 ± 11.27, 65.42 ± 10.42, 67.22 ± 14.16, 45.89 ± 9.76, 42.35 ± 9.54, 54.28 ± 6.60 respectively. Method of one-way analysis of variance was used to estimate sample size. According to pilot study results, the means of six groups were set to 66, 65, 67, 46, 42, 54. And the standard deviation was preset to 14, because the largest SD was 14.16. Significance level was set to 0.05 for two-sided tests (α = 0.05), and the power was set to 0.9 (β = 0.1). Estimation results showed that the sample size of each group should be no less than 7. In the present study, the real sample size of each group was from our actual experiments, and more than 7 animals were used in each group. We have not ruled out any raw data. Consequently, 8 to 11 animals from each group were used for this study, and the number of animals or DRG cells used were indicated in the tables and figure legends, respectively. Similar researches regarding sample size were reported previously [10, 11]. An intraperitoneal injection comprising 1% sodium pentobarbital (50 mg/kg body weight, sigma, USA) was administered to anaesthetize the animals, A 1.1 mm diameter indwelling catheter (PE20, Smith Medical, England), lubricated with paraffin oil was gently inserted into the lightly pinched external urethral orifice, and passed through the urethra into the bladder. A 1.5 cm median incision was made in the lower abdomen. The neck of the bladder and the proximal urethra were bluntly separated using a sterilized cotton swab, and the rear of the bladder neck was bluntly separated exposing the bilateral ureters using an ophthalmic forceps in order to avoid ligation. The bladder was lifted, and the potential lacuna between the back of the urethra and the uterus was opened via blunt and sharp dissection, a 3–0 thread was inserted through it, and the proximal urethra ligated. The degree of tightness of the thread knot can be adjusted and it can be moved along with the surrounding tissue when pulling the indwelling catheter, so that the catheter can be easily removed. Four weeks after the initial surgery, the rats were anaesthetized using an intraperitoneal injection of 25% urethane (1.0 mg/kg). The lower abdomen was cut open and the pelvic cavity entered to expose the bladder. The upper part of the bladder was incised, and a plastic bushing inserted and fastened using purse string suture, after which a micro perfusion pump (Promed-Tech., Bellingham, MA, USA) and a three-way connection pressure sensing device (Nidoc970 urodynamic instrument, Yong xin, China) were connected to the other end of the bushing. The pressure measurement parameters were set to pressure mode, scanning speed of 10 s/div, direct current time-constant, acquisition frequency of 400 Hz, and filtering frequency of 30 Hz. In order to calibrate the zero value, physiological saline at 37 °C was administered into the bladder at 0.3 mL/min, with concomitant digital data collection. If during the low-pressure filling period the bladder contraction interval was shortened and the internal bladder pressure surpassed 15 mm H_2_O, the OAB animal model was considered successfully developed, and further experiments were conducted.

### Determination of urodynamic parameters

In accordance with a published method [12], after having been prevented from drinking but not from eating the night before, the rats were anaesthetized by intraperitoneally administered 25% urethane (1.0 g/kg), and a 24-gauge catheter (Surflo, Terumo Corp., Tokyo, Japan) was buried within the dome of the bladder. A three-channel connector was used to connect the catheter and the pressure sensor (TP-200 T, Nihon Kohden, Tokyo, Japan). The catheter was also connected via a threefold to a pump for continuous administration of physiological saline at 0.1 mL/min. Bladder pressure and urinary volume were recorded by the software on the connected computer during the saline administration process. The measured parameters also included micturition interval (MI), micturition volume (MV), micturition time (MT), pressure difference between the maximal micturition pressure and the basal pressure (MP-BP), and the pressure difference between the threshold pressure and basal pressure [13]. For each animal, 6–8 micturition cycles were recorded, and the average value was taken as the final result [14].

### Electrophysiological characterization of dorsal root ganglia (DRG)

#### Preparation of DRG samples

After anesthesia via intraperitoneal injection of sodium pentobarbital (40 mg/kg), the L6 DRG was exposed and completely separated from surrounding muscle tissue using a glass needle. The extracted DRG was placed into artificial cerebrospinal fluid (ACSF, comprising (mM): NaCl 124, MgCl_2_ 1, KCl 2.5, NaH_2_PO_4_ 1.2, CaCl_2_ 2, NaHCO_3_ 25, Glucose 10, pH 7.4 set using 1 N HCl or NaOH, osmotic pressure set to 290–310 mOsm), which had been air-saturated for 30 min. The upper surface of the specimen was cleaned from connective tissue and covering membranes under a dissecting microscope, after which it was placed into a centrifuge tube containing 0.5 mL of a protease solution comprising 1.0 mg/mL collagenase (Sigma, USA) and 0.4 mg/mL trypsin (Sigma, USA) in PBS pH 7.4, and incubated in a water bath at 37 °C for 40 min. After digestion was completed, the DRG specimen was removed and washed three times with 25 mL ACSF, after which it was incubated in air-saturated ACSF for 1 h [15].

### Whole-cell patch-clamp measurements

The DRG sample was carefully transferred to a perfusion chamber (SHD-27LPKIT, DL Nature gene Life Science, China), fastened using a u-shaped platinum sample holder, and irrigated using a perfusion pump (BT100-2 J/DG-2, Longer, China) at a controlled rate of 1–2 mL/min. An upright microscope was used to investigate the intactness and distribution of cells in the DRG sample, and a cell with a clear 35–40 μm outline and clean surface was selected as the patch-clamp target. A glass electrode (TW150F-4, WPI, USA, electrode resistance 3–6 MΩ) filled with the appropriate amount of electrode solution (mM: Glucose 120, MgCl_2_ 2, KCl 18, CaCl_2_ 1, EGTA 5, HEPES 10, Na_2_-ATP 5, Na_3_-GTP 0.4, pH set to 7.4 using 1 N HCl, osmotic pressure set to 280–300 mOsm) was placed into the electrode holder, after which a 1 mL syringe was used to apply positive pressure to prevent contamination of the electrode tip. As the electrode was slowly moved toward the target cell, an “umbilical” indentation formed. When the electrical resistance in this seal test rose to 0.1–0.3 MΩ, the positive pressure was removed, providing a small amount of negative pressure, forming a high-resistance seal between the cell and the electrode tip. This ensured a GΩ resistance level, which induced the cells to rupture the membrane, thus forming the whole-cell patch clamp. After the clamp was formed, Cm, Vm and Ra were measured. Under the current clamp, the cell’s resting potential was measured, and cells exhibiting a Ra value < 20 MΩ and a resting potential below − 50 mV were selected for further experiments [15].

Under the electrical-current clamp, the DRG neuron was stimulated with a 600 ms depolarizing square wave based on the resting potential, at 100-pA intervals. The induced action potentials (AP) were recorded, and their characteristics analyzed. The first action potential was chosen to determine changes of the AP amplitude, AHP amplitude, threshold value and half width, as well as the membrane potential, Cm and Rm. The amplitude of the AP (mV) was defined as the difference in electric potential between its beginning and highest point, the threshold value (mV) was defined as the membrane potential level at 30% of the rising slope after AP differentiation, the half width (ms) refers to the time difference between two corresponding points on the ascending and descending branches starting at the beginning of the AP, and AHP amplitude denotes the difference in electric potential between the membrane potential and the lowest point of the hyperpolarization. The threshold strength of the medium-size DRG cells (expressed in pA), defined as the smallest current that can elicit an AP from the cells, was recorded under the current clamp. During the experiment, the cells were stimulated with a square-wave stimulus, starting from − 100 pA and rising in steps of 10 pA, with a duration of 60 ms. In order to prevent cell damage, the upper limit of the square-wave stimulus was set to 1000 pA. The 1.5-fold current strength of the rheobase was used to record the number of APs in the medium-size DRG cells, yielding the statistical current strength, with a square-wave stimulus duration of 600 ms. The cells were clamped at − 60 mV, in order to stimulate an *I*_h_, and a hyperpolarization potential was applied starting from − 110 mV and increasing in 10 mV steps to − 60 mV. The slow depolarization constant τ was fitted from *I*_h_ using the formula It = *I*_ss_ + *I*_h_·exp. − t/τ, to obtain the size of the current for time t, whereby *I*_ss_ is the steady-state current value [7].

### Intrathecal injection of ZD7288

Intrathecal cathetererization was conducted according to a published report [16]. Briefly, an incision encompassing 1.5–2.0 cm was made along the spinous processes of the spine, and the muscle tissue separated via blunt dissection. A PE-10 catheter (Smith Medical, England), was slowly inserted with its port plugged into the intervertebral foramen, and fixed with dental cement once it came in contact with the DRG. The control group animals also had PE-10 catheters inserted into the intervertebral foramen and fixed with dental cement. A 1 cm segment of the fixed catheter was exposed. After burying the tube, the animals’ locomotor function was investigated, and individuals with normal mobility were used for further experiments. Three days after surgery, ZD7288 (Sigma, USA) was injected into the exposed catheter (2 μM, in 10 μL of physiological saline, once per day) and continued for 7 days. The control group was injected with the corresponding amount of pure physiological saline. After pharmaceutical administration for 7 days, the urodynamic parameters of the administered group were measured again. Animals were sacrificed via an overdose of sodium pentobarbital at the end of the experiment.

### Statistical analysis

The experimental data are represented as mean ± SD. The patch clamp data were analyzed using the Clampfit software (Version 9.2, Axon, USA), charts were drawn using Origin 8.0, and statistical analysis was conducted using SPSS 16.0 (SPSS, USA). Statistical analyses were performed by with Student’s *t* test when two groups were compared. Results for more than two groups were evaluated by one-way ANOVA followed by Tukey post hoc test. Results were considered statistically significant at *p <* 0.05.

## Results

### ZD7288 significantly reduces urinary bladder weight in OAB rats

The average whole-body and bladder weights of the rats in each group are listed in Table 1. The OAB rats did not show any statistically significant differences in body weight (*p* > 0.05). One week after the OAB rats were intrathecally administered either ZD7288 or saline at the L6 DRG, the body weight also did not change in a statistically significant way (*p* > 0.05). Furthermore, following the experimental endpoint, the urinary bladders of the SD rats were removed, and their weights were found to be significantly increased in the treatment group compared to the sham- group (*p <* 0.01,). Furthermore, this increase was significantly lowered upon ZD7288 administration (*p <* 0.01 compared to the sham- group). Intrathecal administration of saline did not have a significant effect on the bladder weight of the OAB rats (*p* > 0.05, OAB vs OAB + saline).

**Table 1:** Average whole-body and bladder weights of the rats in different groups after intrathecal pharmaceutical administration for 7 days

Parameter	Sham intervention	OAB	OAB + saline	OAB + ZD7288
Body weight (g)	233.21 ± 11.12	229.34 ± 12.13	235 ± 10.67	231.41 ± 13.51
Bladder weight(mg)	121.32 ± 7.56	214.53 ± 11.42**	211.34 ± 12.28	187.38 ± 12.63^##^
n	8	11	11	11

*OAB* Overactive bladder syndrome group; OAB + saline: OAB group treated with saline; OAB + ZD7288: OAB group treated with ZD7288; **p < 0.01, comparison between the OAB and sham-intervention groups; ^##^ *p* < 0.01, comparison between the OAB and OAB + ZD7288 groups (one-way ANOVA)

### Decrease of urinary bladder function in the OAB rats

Table 2 lists the micturition time, micturition interval, micturition volume, the difference between maximal micturition pressure and the basal pressure, as well as the difference between the threshold pressure and basal pressure. Statistical analysis revealed that in the OAB group, the micturition interval was significantly shortened (*p* < 0.01), micturition volume was significantly decreased (*p* < 0.01), and the duration of micturition was significantly longer (*p* < 0.05), compared to the sham group. The difference between the basal and maximal urination pressure, as well as the difference between the threshold pressure and the basic pressure did not change significantly (*p* > 0.05, OAB vs sham). One week after intrathecal administration of ZD7288, the micturition interval and volume of OAB + ZD7288 rats were significantly increased, compared with OAB rats (*p <* 0.01). The micturition time was also significantly reduced (*p <* 0.05, OAB + ZD7288 vs OAB). However, 2 μM ZD7288 did not significantly affect either the difference between the basal and maximal urine pressure, or the difference between the basal and the threshold pressure (*p >* 0.05, OAB + ZD7288 vs OAB). We also investigated the effects of intrathecal administration of saline on voiding function, without discovering significant differences in any of the experimentally determined parameters (*p >* 0.05, OAB vs OAB + saline).

**Table 2:** Urinary bladder and voiding parameters, determined for each group of animals after intrathecal pharmaceutical administration for 7 days

Parameter	Sham intervention	OAB	OAB + saline	OAB + ZD7288
Micturition Interval (Sec)	75.23 ± 5.71	32.85 ± 4.32**	33.45 ± 3.78	48.18 ± 6.58^##^
Micturition Volume (ml)	0.63 ± 0.42	0.21 ± 0.05**	0.34 ± 0.24	0.58 ± 0.32^##^
Micturition Time (s)	10.38 ± 0.74	12.65 ± 1.31*	13.02 ± 1.54	10.54 ± 1.53^#^
Difference between the Maximal and Basal Micturition Pressures (cmH_2_O)	54.35 ± 4.67	52.28 ± 7.49	51.76 ± 6.98	49.53 ± 4.87
Difference between the Threshold Pressure and Basal Pressure (cmH_2_O)	12.86 ± 1.08	9.59 ± 6.75	9.78 ± 7.31	8.87 ± 7.21
n	8	11	11	11

*OAB* Overactive bladder syndrome group; OAB + saline: OAB group treated with saline; OAB + ZD7288: OAB group treated with ZD7288; *p < 0.05, **p < 0.01, OAB group compared to the sham-intervention group; ^#^*p* < 0.05, ^##^*p* < 0.01, OAB group compared to the OAB + ZD7288 group (one-way ANOVA)

### Increase of excitability of the medium-size DRG neurons in the OAB rats

In the whole-call patch clam experiment, we recorded the resting membrane potential (RMP), cell capacitance (Cm), and input resistance (R_in_) of the medium-size DRG neurons. As is shown in Table 3, the RMP and R_in_ of the OAB rats were significantly decreased (*p* < 0.05), while the cells’ radius and Cm did not exhibit significant changes compared with the sham group (*p* > 0.05).

**Table 3:** Passive electrophysiological characteristics of the middle-size DRG neurons in the OAB and sham-intervention groups

Parameter	Sham intervention	OAB
Cell size (μm)	41.36 ± 1.34	42.21 ± 1.25
RMP(mV)	−58.49 ± 1.72	−51.03 ± 2.45*
R_in_ (MΩ)	54.87 ± 5.36	49.38 ± .54*
C_m_ (pF)	63.41 ± 6.37	65.39 ± 7.82
n	24	19

*OAB* denotes the overactive bladder syndrome group, n represents the number of individual investigated cells, *RMP* is the resting membrane potential, R_in_ is input resistance, and C_m_ is cell capacitance. Comparison of DRG in the OAB and sham-intervention groups revealed no significant difference in C_m_ (*p* > 0.05, Student’s *t* test), while the RMP was significantly less negative and R_in_ was significantly decreased in the OAB group (*p < 0.05 for both, Student’s *t* test)

**Fig. 1:**
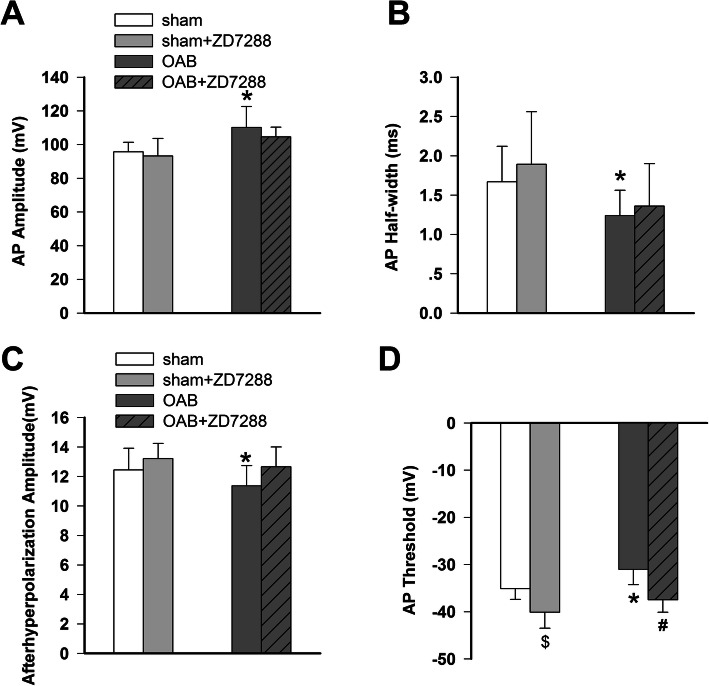
Effects of ZD288 on the AP amplitude, half-width, threshold value, and after hyperpolarization amplitude of the medium-size DRG neurons in OAB and sham-intervention rats. The amplitude, half-width, afterhyperpolarization potential (AHP) amplitude and threshold value of the first induced action potential (AP) of the medium-size DRG cells in the OAB rat model were investigated, together with the effect of ZD7288 (2 μM) administration on said parameters. **a**: the amplitude of the action potential was significantly increased in OAB rats, **b**: the action potential half-width was significantly reduced in OAB rats, **c**: the amplitude of the afterhyperpolarization potential was significantly reduced in OAB rats, **d**: the action potential threshold value of OAB rats was significantly lowered; ZD7288 significantly reduced the threshold value of both OAB and sham-intervention rats, but did not affect the AP amplitude, half-width, and AHP amplitude. (*p < 0.05, sham vs OAB group, ^#^*p* < 0.05, OAB vs OAB + ZD7288 group,; ^$^*p* < 0.05, sham vs sham+ZD7288 group,; sham *n* = 24, sham+ZD7288 *n* = 24, OAB *n* = 19, OAB + ZD7288 *n* = 19

**Fig. 2:**
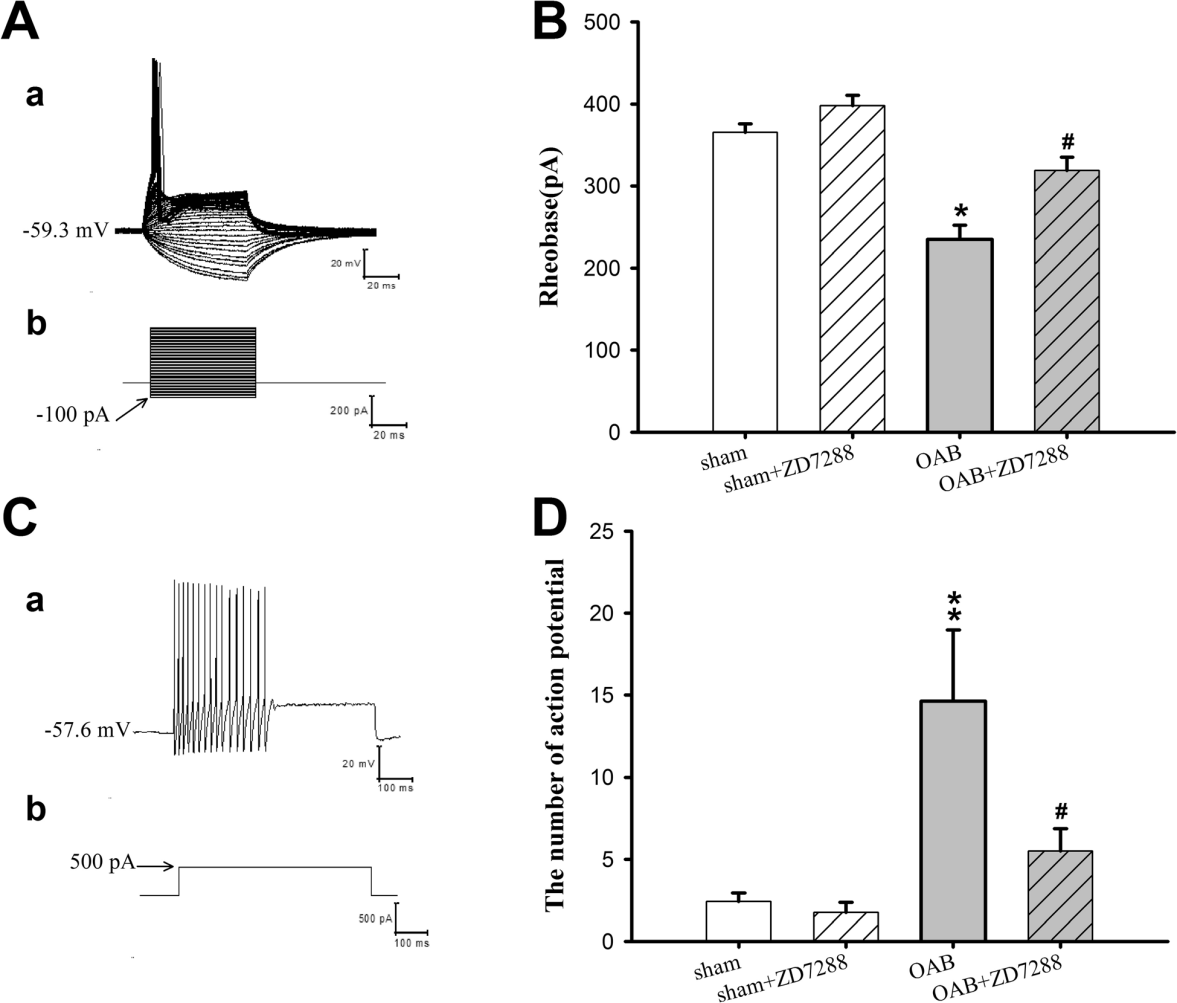
Effects of ZD7288 on the threshold value and number of action potentials of the medium-size DRG cells in OAB and sham-intervention rats. The threshold value and number of induced action potentials (AP) of medium-size DRG cells in the rat model of OAB and the effect of ZD7288 on both parameters were investigated. **a**:a shows the diagram of the original action potentials produced by the medium-size DRG cells, b shows the square wave used to induce the APs (starting from − 100 pA, increasing in steps of 10 pA); **b**: the threshold intensity of the cells in the OAB group was significantly decreased, and was significantly increased by 2 μM ZD7288; **c**: a shows the original diagram of the medium-size DRG cells’ action potential, b shows the 500 pA square wave used to elicit the action potentials; **d**: statistical analysis of the number of APs revealed a significant increase in the OAB group, which was significantly decreased by ZD7288 (*p < 0.05, ** *p* < 0.01, sham vs OAB group; ^#^*p* < 0.05, OAB vs OAB + ZD7288 group; sham *n* = 24, sham+ZD7288 *n* = 24, OAB *n* = 19, OAB + ZD7288 *n* = 19)

Thus, the passive membrane characteristics of the medium-size DRG cells did not change significantly under OAB conditions. However, did the active characteristics change? Compared with the sham group, the AP amplitude of the medium-size DRG neurons in the OAB group was significantly increased (from 95.64 ± 5.70 mV to 110.21 ± 12.34 mV), and the half-width decreased from 1.67 ± 0.45 ms to 1.24 ± 0.32 ms (Fig. 1c), the afterhyperpolarization amplitude decreased from 12.45 ± 1.45 mV to 10.37 ± 1.37 mV (Fig. 1c), and the threshold value was significantly less negative (− 31.03 ± 3.24 mV compared to − 38.10 ± 2.31 mV in the control, Fig. 1d) with *p* values < 0.05 in all cases (OAB vs sham).Taken together, these results indicate that the excitability of the medium-size DRG neurons of the OAB rats was significantly increased. Furthermore, we investigated the lowest stimulus strength of the current (equal to the rheobase) capable of eliciting an AP from the medium-size DRG neurons. The results show that the rheobase was significantly decreased in the OAB group (365.36 ± 10.54 pA in sham vs 234.67 ± 17.45 pA in OAB, *p* < 0.05, Fig. 2a).

In order to quantify the cells’ excitability, we measured the number of action potentials (APs) induced by a current corresponding to the 1.5-fold of the rheobase. The results thus represent the number of APs induced by a current of 500 pA, and the number of APs induced in cells from the OAB group was significantly higher than in the sham group (14.63 ± 4.35 in OAB vs 2.42 ± 0.51 in sham, *p* < 0.01, Fig. 2c and d).

### ZD7288 significantly decreased the excitability of the medium-size DRG cells in the OAB rats

Taken together, the above-stated results indicate that the excitability of the medium-size DRG cells increased in the OAB rats. Our research further revealed that an intrathecal injection of ZD7288 was able to significantly decrease the OAB rats’ bladder weight as well as to significantly improve their voiding function (Table 2). This in turn means that ZD7288 can influence the excitability of the DRG neurons, reducing the DRG-mediated activation of the bladder, and thus lowering bladder motility. The *I*_h_ current induces the slow depolarization of the cell membrane at the beginning of the AP, so that, after the AP burst and until the hyperpolarization potential, there is an inactivation of the *I*_h_ load-bearing channel. Therefore, the *I*_h_ current can influence the AP threshold value, which is consistent with the finding that the threshold value was significantly increased by ZD7288 in both the OAB and sham groups (− 35.03 ± 2.31 Mv in sham vs − 40.10 ± 3.41 mV in sham+ZD7288; − 31.03 ± 3.24 Mv in OAB vs − 37.46 ± 2.66 mV in OAB + ZD7288, *p* < 0.05 in both cases; Fig. 1d). The rheobase of the medium-size cells in the L6 DRG ganglia of the OAB animals was also significantly increased by ZD7288 (234.67 ± 17.45 pA in OAB vs 318.82 ± 16.39 pA in OAB + ZD7288, *p* < 0.05; Fig. 2b). Additionally, the experiments revealed that the number of APs induced by a 500-pA current was significantly decreased in the OAB + ZD7288 group (14.63 ± 4.35 in OAB vs 5.50 ± 1.37 in OAB + ZD7288, *p* < 0.05; Fig. 2d).

However, ZD7288 did not have any significant effect on the amplitude of the afterhyperpolarization potential or half-width (*p* > 0.05, OAB + ZD7288 vs OAB and sham+ZD7288 vs sham; Figs. 1a, b and c).

**Fig. 3:**
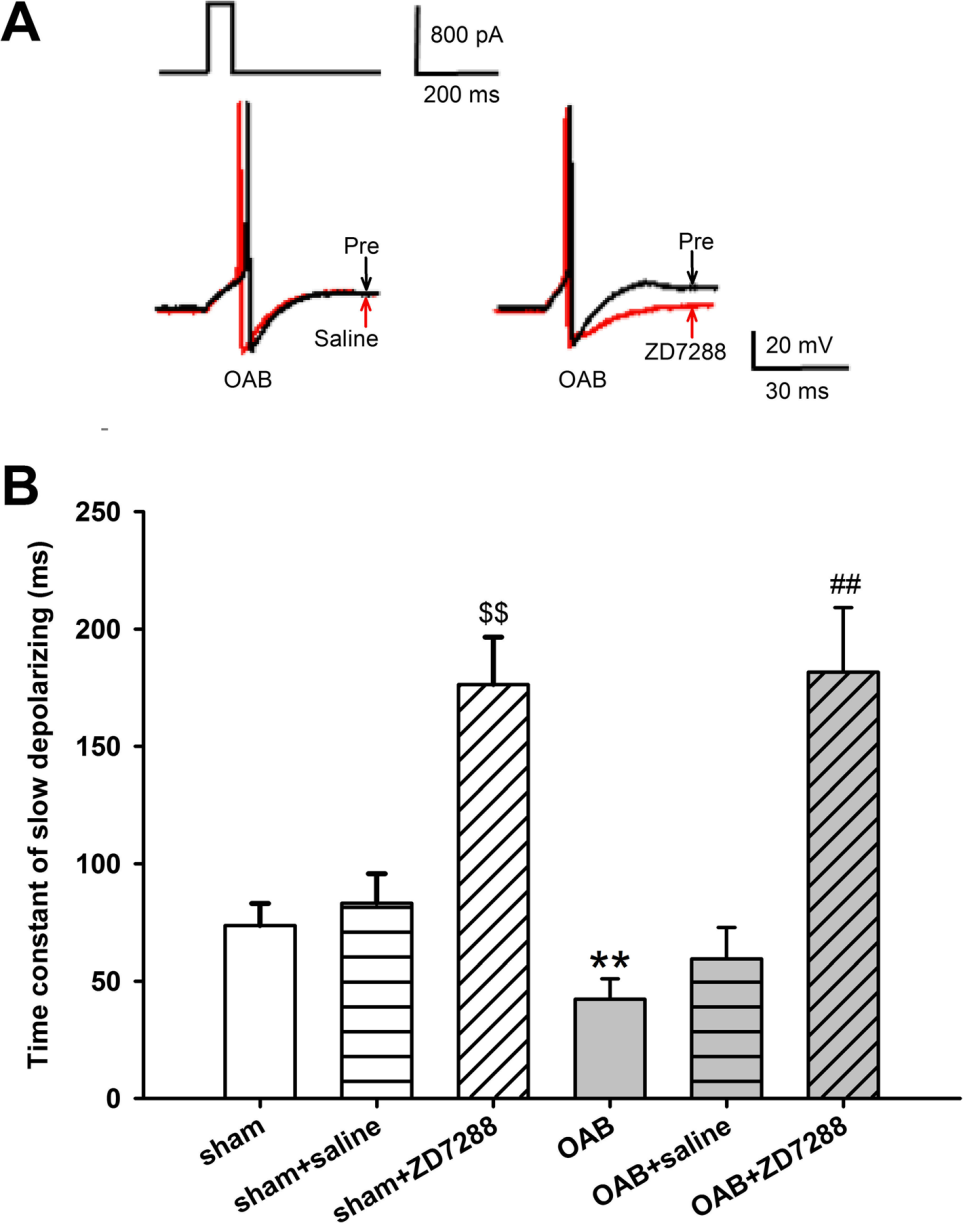
Effects of ZD7288 on the τ value of the medium-size cells in the L6 DRG of the OAB and sham-intervention rats. The τ value (time constant of slow depolarizing) of the DRG medium-size cells in the DRG of the OAB model rats was investigated, along with the effect of ZD7288 on this parameter. **a**: Square-wave stimulation of DRG medium-size cells under current-clamp conditions (800 pA, 60 ms, first row). ZD7288 significantly increased the τ value in the OAB model, whereas saline did not have an effect. **b**: Change of the τ value. The τ value was significantly decreased in the OAB rats, and ZD7288 was able to significantly increase it in both OAB and sham groups **(**^**$$**^*p* < 0.01, sham vs sham+ZD7288 group; **p < 0.01, sham vs OAB group; ^##^*p* < 0.01, OAB vs OAB + ZD7288 group; sham *n* = 12, sham+ZD7288 *n* = 12, OAB *n* = 13, OAB + ZD7288 *n* = 13)

During the AP hyperpolarization process, the *I*_h_ current is activated and involves in the recovery of the membrane following the AHP [17, 18]. This means that the *I*_h_ current may possibly influence the time-course of the recovery of the hyperpolarization potential towards the level of the membrane potential. The experiments have uncovered that the τ value in the OAB model was significantly reduced (73.7 ± 9.4 ms in sham vs 42.35 ± 8.67 ms in OAB; *p* < 0.01; Fig. 3b), indicating that the excitability of the OAB rats’ medium-size DRG cells increased, which was in agreement with the increase of the number of preceding discharges. ZD7288 (2 μM) was able to significantly increase the τ values of both the OAB and sham animals (73.7 ± 9.4 ms in sham vs 176.34 ± 20.30 ms in sham+ZD7288; 42.35 ± 8.67 ms in OAB vs 181.56 ± 27.45 ms in OAB + ZD7288; *p* < 0.01, in both cases; Fig. 3b).

At the same time, saline administration did not have a significant effect on the τ value of either the OAB or the sham group (73.7 ± 9.4 ms in sham vs 83.26 ± 12.56 ms in sham+saline; 42.35 ± 8.67 ms in OAB vs 59.44 ± 13.43 ms in OAB+ saline; *p* > 0.05 in both cases; Fig. 3b).

### Measurement of *I*_h_ current density in the medium-size DRG cells of the OAB rats with ZD7288 treatment

**Fig. 4:**
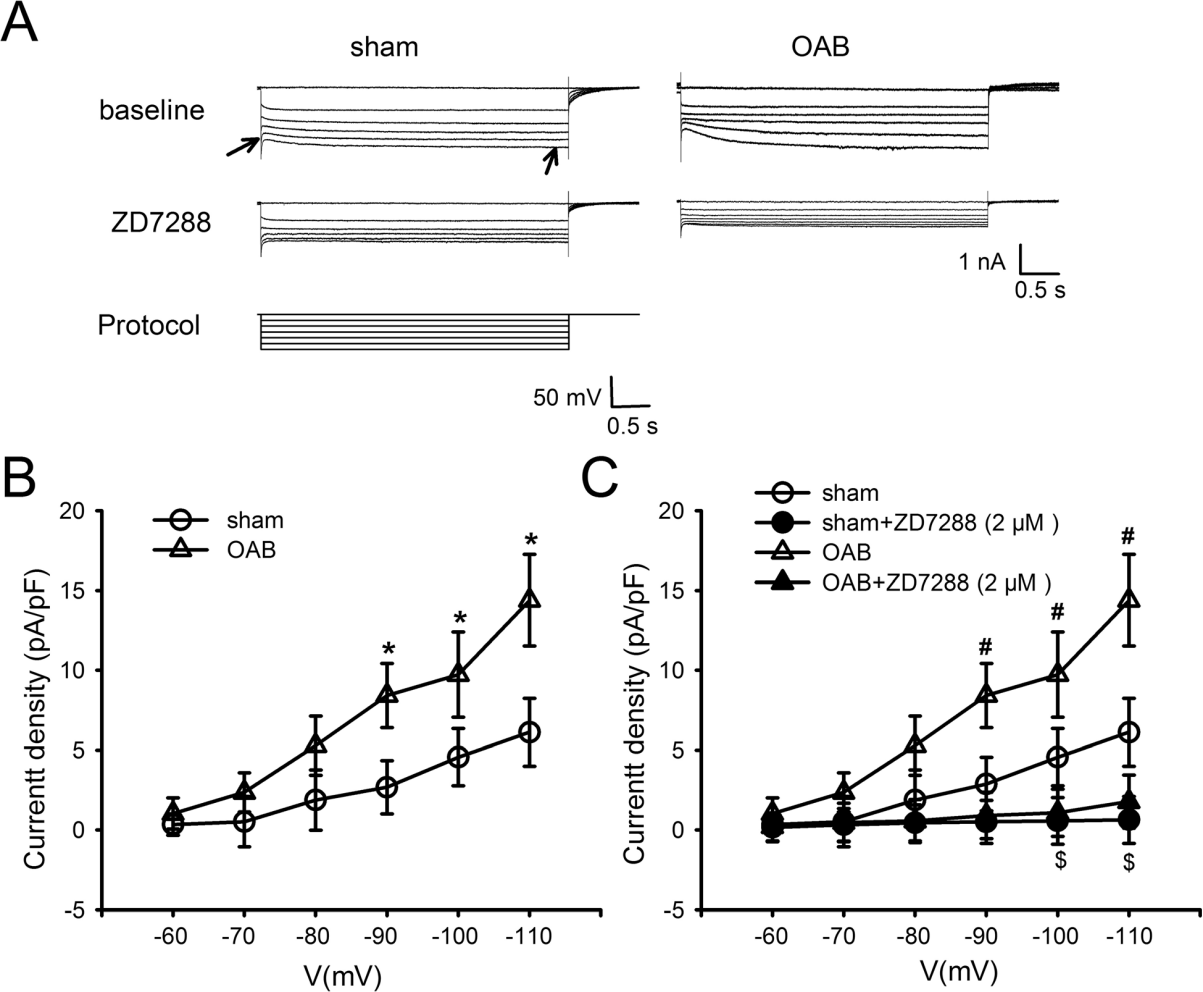
Measurement of *I*_h_ current density in the medium-size DRG cells of the OAB and sham-intervention rats. **a**: The *I*_h_ current eliciting a hyperpolarization potential in the medium-size DRG cells of the sham group (left side) and OAB group (right side). The magnitude of the *I*_h_ current is the current difference corresponding to the vertical distance from the instantaneous starting point to the steady state (the two arrows in the diagram indicate the endpoints of the magnitude). The ratio of the *I*_h_ amplitude and the capacitance of the neural cells is the *I*_h_ current density. After the addition of 2 μM ZD7288 to the perfusion fluid, the *I*_h_ current of both the OAB and sham groups was significantly inhibited (middle row). The final row shows the *I*_h_ current induced by the square-wave stimulus (starting from − 110 mV, increasing in 10 mV steps). **b**: When said cells were clamped at − 110, − 100 and − 90 mV, the *I*_h_ current density was significantly increased. C: The *I*_h_ current density of the both the OAB and sham groups was significantly reduced by ZD7288 (*p < 0.05, sham vs OAB group; #*p* < 0.05, OAB vs OAB + ZD7288 group; $*p* < 0.05, sham vs sham+ZD7288 group; sham *n* = 11, sham+ZD7288 *n* = 11, OAB *n* = 13, OAB + ZD7288 *n* = 13)

The above-described experiments revealed that ZD7288 is able to significantly reduce the threshold value, threshold intensity, and number of APs, in addition to significantly increasing the τ value of the L6 DRG neurons in the OAB rats. These results thus indicate that the formation of the *I*_h_ current in the OAB model might play an inordinately important role. In the voltage clamp experiments, the cells were clamped at − 60 mV, and the hyperpolarization potential was increased in 10 mV steps from − 110 mV to − 60 mV, inducing a slow inward cation current (Fig. 4a). Furthermore, this type of hyperpolarization-activating inward current could be completely blocked by 2 μM ZD7288, confirming that the recorded current is indeed the *I*_h_ current. When the potential was clamped at − 90, − 100 and − 110 mV, the *I*_h_ current density was significantly higher in OAB group compared with control (at − 90 mV: 2.68 ± 1.67 pA/pF in sham vs 8.43 ± 2.10 pA/pF in OAB; at − 100 mV: 4.56 ± 1.79 pA/pF in sham vs 9.73 ± 2.67 pA/pF in OAB; at − 110 mV: 6.12 ± 2.13 pA/pF in sham vs 14.39 ± 2.87 pA/pF in OAB; *p* < 0.05, Fig. 4b). In contrast, 15 min after 2 μM ZD7288 was added to the measurement buffer in which the cells were immersed, the *I*_h_ current density of the DRG neurons in the OAB group was significantly lower (at − 90 mV, 8.43 ± 2.10 pA/pF in OAB vs 0.91 ± 1.45 pA/pF in OAB + ZD7288; at − 100 mV, 9.73 ± 2.67 pA/pF in OAB vs 1.08 ± 1.48 pA/pF in OAB + ZD7288; at − 110 mV, 14.39 ± 2.87 pA/pF in OAB vs 1.78 ± 1.67 pA/pF in OAB + ZD7288; *p* < 0.05, Fig. 4c).

During the same experiment, it was discovered that ZD7288 was able to significantly reduce the *I*_h_ current density of the DRG neurons in the sham rats (at − 100 mV: 4.56 ± 1.79 pA/pF in sham vs 0.57 ± 1.46 pA/pF in sham+ZD7288; at − 110 mV: 6.12 ± 2.13 pA/pF in sham vs 0.64 ± 1.47 pA/pF in sham+ZD7288; *p* < 0.05; Fig. 4c). However, the reduction range of current density of DRG neurons after ZD7288 administration were obviously wider in OAB rats than sham rats (Fig. 4c). These results suggested that ZD7288 could inhibit cell excitability of DRG cells relatively more strongly in OAB group than in sham group.

## Discussion

The urinary bladder weight of the overactive bladder syndrome (OAB) model rats, as well as the excitability and *I*_h_ current density of the animals’ medium-size DRG neurons were significantly increased. Intrathecal injection of the specific *I*_h_ current inhibitor ZD7288 was able to alleviate the observed changes in the OAB rats, including a prolonged micturition time and shortened micturition interval. At the same time, ZD7288 completely blocked the *I*_h_ current density, and lowered the excitability of the medium-size DRG cells. Taken together, these results strongly suggest that the *I*_h_ current may be a factor in the development of OAB.

The partial bladder outlet obstruction rat model is a good approximation of the condition seen in clinical patients with urinary bladder overactivity and urethral blockage syndromes, including a high degree of agreement in the observed pathological changes [19]. Therefore, the partial bladder outlet obstruction rat model was used in this study, and the urinary bladder weight of the successfully induced OAB rats was significantly increased, in agreement with the literature [13]. Recordings of the micturition process have uncovered that the micturition interval of the OAB rats was significantly shortened, their micturition time was significantly increased, and micturition volume was significantly decreased, indicating that the rats with the partially-blocked urethra indeed had an abnormally enhanced micturition reflex.

According to anatomical studies, the neural fibers that innervate the bladder include the myelinated A_δ_ and the myelin-free C-type fibers [6, 19, 20]. One part of these afferent fibers transverses the pelvis, arriving at the L6 and S1 DRG of the spine, and takes part in the initial stage of the micturition signal, while another part transverses the lower abdomen, arriving at the L1 and L2 level of the spine [3, 21, 22]. The DRG neurons are classified into small, medium and large types according to their cross-sectional diameter, and further separated according to the A_α/β_, A_δ_ and C typology [4, 5]. A subset of the nerve fibers that control the micturition process terminate at the L6 DRG, making the question pertinent whether the excitability of the OAB rats’ L6 DRG has exhibited any changes. The results of present study indicated that the AP amplitude and number of APs elicited by a 500-pA stimulus were significantly increased, whereas the half-width, threshold strength, threshold value and the afterhyperpolarization potential, as well as the input resistance were significantly decreased. The membrane potential also had an obvious depolarization tendency. All these results suggested that the medium-size L6 DRG cells of the OAB rats had a significantly increased excitability. The membrane potential of the L6 DRG neurons of the OAB rats also displayed obvious depolarization, with an increase in the number of APs elicited by a 500-pA stimulus, as well as a decrease of the input resistance and τ value. Thus, the hyperpolarization-induced cation current (*I*_h_) might play an important role in the observed increased excitability of the medium-size neurons in the L6 DRG of the OAB rats. The *I*_h_ current is present in excitable cells in both the center and the periphery of the body, including the photoreceptor cells of the retina and the cardiac pace-maker cells, as well as cells of the central and peripheral nervous system [23, 24]. The *I*_h_ current channel is activated when the AHP is rapidly increasing, and the influx of Na^+^ causes a slow depolarization of the cell membrane. In peripheral nerves, *I*_h_ is found in the sensory DRG neurons [25]. Within these sensory neurons, it participates in the AHP process, and induces the cell membrane to recover to the resting potential after depolarization, shortening the AHP amplitude and duration [26, 17, 27]. Studies have also reported that the *I*_h_ current plays a role in the maintenance of membrane potential, influencing the cells’ spontaneous and repeated discharge, as well as related phenomena [28, 29]. In our experiments, it was shown that the specific *I*_h_ current inhibitor ZD7288 is able to clearly inhibit the excitability of the medium-size DRG cells in the OAB rats, including a reduction of the number of APs elicited by a 500-pA stimulus and an increase of the τ value, indicating that the *I*_h_ current within the L6 DRG neurons plays an important role in the etiology of OAB. After administration of ZD7288, DRG in sham+ZD7288 group also showed elevated τ value and reduction in current density, suggested that *I*_h_ current inhibitor could reduce the cell membrane excitability in sham rats. However, the reduction range of current density of DRG neurons after ZD7288 administration were obviously wider in OAB + ZD7288 group than sham+ZD7288 group. These results suggested that ZD7288 could inhibit cell excitability of DRG cells relatively more strongly in OAB rats than in sham rats.

A study by Masuda and coworkers has found that the *I*_h_ current is primarily present in the medium-size DRG neurons that control the urinary bladder, and that inhibition of the current significantly increases the time from the highest AHP point until the return to the resting potential, and reduces the excitability of the medium-size DRG neurons [7]. These results imply that the excitability of the medium-size mechanical nociceptor cells in the DRG that control the urinary bladder may be influenced by the *I*_h_ current. This raises the question whether the *I*_h_ current in the medium-size DRG cells of the OAB mice experienced changes. Our results show that the *I*_h_ current density was significantly increased, and functional experiments have shown that intrathecal injection of ZD7288 significantly improved the micturition parameters of the OAB rats. The experimental results indicate that an increase in the strength of the *I*_h_ current in the medium-size cells of the L6 DRG might be an important component of the OAB development process. Furthermore, the hyperpolarization-activated cyclic nucleotide-gated cation channel (HCN) mediates the *I*_h_ current, and an investigation of HCN expression in the neurons that control the bladder, which are found in the L6-S1 DRG, has uncovered rich expression of HCN2 in the average medium-size DRG cells.

It is thus theoretically possible that the significant increase of the *I*_h_ current in the medium-size neurons of the L6 DRG of OAB rats is related to the HCN2 channel, and this notion merits further research. Furthermore, these experimental results demonstrate that the half-width, amplitude, and afterhyperpolarization potential of the action potential are not influenced by ZD7288, which is in agreement with other studies, which have also found that ZD2788 cannot influence the shape of the AP or the AHP amplitude [28, 29]. This indicates that the observed changes of the *I*_h_ current may be part of the electrophysiological mechanism responsible for the increase of excitability in the medium-size DRG cells of the OAB rats. The limitations of current study is that the precise functional activity of the *I*_h_ current in the medium-size DRG neurons of OAB rats has not been fully unveiled, and we do not observe the small-size DRG neurons. Further studies using structurally dissimilar HCN channel blockers such as ivabradine will be needed to strengthen our present behavioral and electrophysiological findings. Further electrophysiological mechanisms influencing the cells’ excitability still remain to be resolved. Since the etiology of OAB is complicated and multifactorial, additional studies are needed in different animal models and different OAB related cells to clarify the real role of *I*_h_ current in bladder dysfunction.

## Conclusions

This study reveals that the excitability of medium-size cells in the L6 DRG of OAB model rats was significantly increased, and that the *I*_h_ current correlates with a part of the parameters of this heightened nerve-cell excitability. The medium-size cells in the L6 DRG of the OAB rats had a significantly increased *I*_h_ current density, and their micturition reflex was significantly improved under the influence of ZD7288, which indicates that the *I*_h_ current in these cells may be an important part of the electrophysiological mechanism leading to the development of OAB.

## Data Availability

The datasets used and/or analyzed during the current study are available from the corresponding author on reasonable request.

## Abbreviations

OAB: OVERACTIVE bladder syndrome
DRG: Dorsal root ganglia
MI: Micturition interval;
MV: Micturition volume
MT: Micturition time
MP-BP: Maximal micturition pressure and the basal pressure
HCN: Hyperpolarization-activated cyclic nucleotide-gated cation
RMP: Resting membrane potential
AP: Action potentials
RMP: Resting membrane potential
Cm: Cell capacitance
